# Influence of pH, Temperature and Protease Inhibitors on Kinetics and Mechanism of Thermally Induced Aggregation of Potato Proteins

**DOI:** 10.3390/foods10040796

**Published:** 2021-04-08

**Authors:** David J. Andlinger, Pauline Röscheisen, Claudia Hengst, Ulrich Kulozik

**Affiliations:** Food and Bioprocess Engineering, TUM School of Life Sciences, Technical University of Munich, Weihenstephaner Berg 1, 85354 Freising, Germany; pauline.roescheisen@tum.de (P.R.); claudia.hengst@tum.de (C.H.); ulrich.kulozik@tum.de (U.K.)

**Keywords:** patatin, thiol, sulfhydryl group, hydrophobic, non-linear regression, denaturation, protease inhibitor proteins, PRODAN

## Abstract

Understanding aggregation in food protein systems is essential to control processes ranging from the stabilization of colloidal dispersions to the formation of macroscopic gels. Patatin rich potato protein isolates (PPI) have promising techno-functionality as alternatives to established proteins from egg white or milk. In this work, the influence of pH and temperature on the kinetics of PPI denaturation and aggregation was investigated as an option for targeted functionalization. At a slightly acidic pH, rates of denaturation and aggregation of the globular patatin in PPI were fast. These aggregates were shown to possess a low amount of disulfide bonds and a high amount of exposed hydrophobic amino acids (S_0_). Gradually increasing the pH slowed down the rate of denaturation and aggregation and alkaline pH levels led to an increased formation of disulfide bonds within these aggregates, whereas S_0_ was reduced. Aggregation below denaturation temperature (T_d_) favored aggregation driven by disulfide bridge formation. Aggregation above T_d_ led to fast unfolding, and initial aggregation was less determined by disulfide bridge formation. Inter-molecular disulfide formation occurred during extended heating times. Blocking different protein interactions revealed that the formation of disulfide bond linked aggregation is preceded by the formation of non-covalent bonds. Overall, the results help to control the kinetics, morphology, and interactions of potato protein aggregation for potential applications in food systems.

## 1. Introduction

Protein aggregates can be used as functional ingredients in different food formulations [1]. The formation of these aggregates can be induced by the unfolding of the globular molecular structure at elevated temperatures. Upon unfolding, proteins lose their tertiary and secondary structure, and reactive amino-acid groups get exposed, and the proteins thus can interact with each other to form aggregates. These interactions can be of covalent or non-covalent nature. The most important covalent bonds are disulfide bonds, which can be formed by the reaction of two thiol-groups or by the reaction of a thiol group with an intramolecular disulfide bond leading to a chain reaction, the thiol-disulfide interchange [2]. Non-covalent interactions comprise hydrophobic interactions and electrostatic interactions. These interactions are weaker than disulfide bonds. The type of protein interaction influences the morphology of the aggregates and is pH and ionic-strength dependent [3].

In comparison to other globular proteins, in particular β-lactoglobulin (β-lg), the major whey protein from bovine milk, the aggregation mechanism has been much less well studied and understood for plant proteins, including patatin, the main protein from the potato tuber. Patatin has a molecular mass of around 40–42 kDa, and four different isoforms are known [4]. The isoelectric point (IEP) of patatin is between a pH of 4.5–5.1 [5]. It comprises around 40% of the total potato protein [6]. The heat-induced unfolding of patatin at pH 8 was shown to be partly reversible [7]. It was shown that initial unfolding occurs at temperatures as low as 28 °C. α-helixes unfold between 45–55 °C followed by β-strands between 50–90 °C. This unfolding was accompanied by precipitation. Like for other globular proteins, heat-induced patatin aggregation was shown to follow a two-step aggregation scheme where the native protein first reversibly unfolds and then irreversibly forms a reactive species, which can form larger aggregates [8]. At temperatures at and above 60 °C, no unfolding reaction rate constant could be determined, probably because it happened too fast. The authors concluded that detailed insights into this physical reaction step that preceded aggregation could not be deduced. Another study found that heating of patatin resulted in the formation of dimers and trimers [9]. The disintegration of these structures following the addition of β-mercaptoethanol indicated that the aggregates were stabilized by disulfide bonds. However, these aggregates also formed in the presence of the thiol blocking agent NEM. The authors postulated that the formation of disulfide links might play a secondary role and hypothesized that disulfide formation might only occur after aggregation through interactions further along the thermal process. However, no time-resolved analysis on the reactivity of the thiol groups was performed. Another study compared the aggregation kinetics of patatin to two other major food proteins, i.e., ovalbumin from egg white and β-lg from whey [10]. Higher aggregation rates were found for ovalbumin and patatin in comparison to β-lg. However, this order of aggregation rates could not be explained by simple molecular differences such as exposed hydrophobicity, free sulfhydryl groups, or net surface charge density. Furthermore, the kinetic data showed differences between β-lg and patatin. In contrast to β-lg, different heating conditions could not be combined in a simple denaturation scheme for patatin. Certain heating conditions clearly deviated from the averaged denaturation curve, a fact that could not be fully explained by the data. There was a clear distinction between conditions of rapid denaturation above T_d_ and slow denaturation below T_d_. Furthermore, there were differences between the electrostatic repulsion at neutral pH and at reduced pH, closer to the IEP. Therefore, more research appears to be required to better understand the aggregation mechanism under a wider range of heating conditions.

Aggregation and gelation of patatin were described by several studies as driven by hydrophobic interactions. Although the hydrophobicity of unheated patatin has been compared to β-lg and ovalbumin, the results are not conclusive. Patatin was found to be more hydrophobic than β-lg when investigated by hydrophobic interaction chromatography [11] and less hydrophobic when investigated by the fluorescence dye ANS (1-anilinonaphthalene-8-sulfonic acid) [10]. Furthermore, the hydrophobicity of aggregated patatin has not been assessed so far.

After patatin, the second most abundant group of potato proteins are protease inhibitors (PI). PI are a heterogeneous group of proteins with IEPs between 5–8 and molecular weights between 7–21 kDa [12,13]. The first and second most abundant protease inhibitors are potato serine protease inhibitors [14] and potato cysteine protease inhibitors [15], respectively. From both of these PI, it is known that their thermal stability is higher than patatin’s, indicated by a higher temperature of unfolding. PIs were shown to form gels, although, at neutral or mildly acidic pH, the gels were very brittle compared to patatin [16]. Besides this, little is known about their techno-functionality. To the best of our knowledge, there is no study investigating if protease inhibitors interact with patatin upon heating.

Although there were already some investigations reported on the aggregation of patatin, the mechanism behind the aggregation, especially in regard to protein interactions, remains unclear. In this study, commercial patatin enriched potato protein isolate (PPI) was used to investigate the heat-induced aggregation of the potato protein patatin and possible interactions with PI. The following approach was chosen: The formation of covalent disulfide bonds was investigated by SDS-PAGE. Certain blocking substances were used to differentiate between the roles of hydrogen, hydrophobic, and thiol interactions in the aggregation of PPI. Blockers, such as sodium dodecyl sulfate (SDS) and N-Ethylmaleimide (NEM), urea, and Tween 20 disrupt interactions with proteins in different ways and block the formation of certain protein interactions [17].

Potato protein denaturation and aggregation were investigated over a broad range of pH values. Changes in pH were applied to change the interactions between the protein monomers during unfolding and aggregation. Protein interactions closer to the isoelectric point (IEP), i.e., at less intense electrostatic interaction between patatin monomers, were studied to assess aggregation by short-ranged bonds. Higher pH values were established to favor the formation of disulfide bonds.

The influence of pH will lead to different properties of the aggregates, such as exposed hydrophobicity and stabilization through disulfide bonds. Such aggregates can be used to stabilize foams [18] and emulsions [19] or as oil-structuring agents [20]. Furthermore, aggregates are considered the building block of protein gels. Depending on the aggregate properties, the properties of the gels can be severely influenced. Such gels can be used as fat replacers [21] and encapsulation systems [22,23], among others. To predict how PPI will behave in these applications, a deeper understanding of the denaturation and aggregation mechanism should be established. Therefore, the aim of this study is to understand these mechanisms in dependence of different heating conditions.

## 2. Materials and Methods

### 2.1. Materials and Sample Preparation

All weight ratios are given as weight percentage (gram protein per 100 g solution) abbreviated as % (*w*/*w*).

Commercial PPI powder (Solanic 200^®^) with a high content of patatin, was obtained from AVEBE (Veendam, The Netherlands). The protein powder had a protein content of 88.6% (*w*/*w*). The protein content was determined using the method of Dumas with an accuracy of ±0.1% (*w*/*w*) and a Dumas factor of 6.25 (Vario MAX CUBE, Elementar Analysen-Systeme GmbH, Hanau, Germany). This conversion factor was already used by other researchers for PPI [16,24] The patatin content, measured by the relative band intensity (RBI) in a reducing SDS-PAGE, was found to be around 60% RBI. A similar patatin concentration for this PPI was reported by others [25]. Other protein components were protease inhibitors (25% RBI) and a higher molecular weight fraction with 100 kDa (15% RBI).

The powder was solubilized in deionized water and stirred overnight, to ensure complete dissolvement of the protein powder. To limit bacterial growth, stirring was done at 4 °C. The water was deionized in a two-step process. Water was softened by removing calcium carbonate followed by deionization by an ion exchange gel. The solution was then centrifuged at 6000× *g* for 15 min to eliminate insoluble components, around 5% of the total protein. The protein concentration of the final solution was determined by using the method of Dumas, similar to the determination for the protein powder. The protein concentration of the final solutions was around 1% (*w*/*w*) (accuracy from a triplicate 0.97 ± 0.04 (*w*/*w*)). In order to investigate the influence of pH on aggregation, the solutions were adjusted with 1 mol/L HCl and 1 mol/L NaOH (Merck, Darmstadt, Germany) to pH 6.0, 7.0, 8.0, 9.0, and 10.0. pH values were checked with a pH meter a deviation of ±0.01 from the target value was deemed sufficient.

### 2.2. Modulated Differential Scanning Calorimetry (mDSC)

The denaturation temperatures (T_d_) of PPI solutions at pH 6.0, 7.0, 8.0, 9.0, and 10.0 were measured using Tzero-calibrated modulated differential scanning calorimetry (mDSC Q1000, TA Instruments, Newcastle, UK). To measure each solution with an individual pH, 20 μL of the solution was filled into a hermetically sealed aluminum pan. As a reference, an empty aluminum pan was used. The temperature was gradually heated from 25 to 90 °C with a modulated heating rate of 2 °C/min. The respective T_d_ for each pH level was used to determine heating temperatures (see Table 1). One example of an mDSC thermogram is given under Supplementary Materials Figure S1.

### 2.3. Heating Experiments

3.14 mL of the protein solution was filled into stainless steel tubes, with a length of 240 mm, an inner diameter of 5 mm, and an outer diameter of 6 mm, closed with a screw cap. Several tubes were prepared this way, transferred into a water bath, and removed from the water bath after a certain heating time intervals to obtain time-resolved information about the aggregation process (see Supplementary Materials Table S1 for an overview of the time intervals). After the heating step, the tubes were immediately put into ice water to stop the heat-induced reaction. The heating temperature chosen was dependent on T_d_ determined by modulated differential scanning calorimetry (mDSC). The denaturation temperature results for pH 6 to 10 [26] are summarized in Table 1. Four temperatures were investigated per pH value. Temperatures for the heating trials were set 5 and 10 °C above and below the determined T_d_. By using this heating regime, similar unfolding kinetics should be achieved. This approach was already used for the comparison of the aggregation behavior of different protein sources [10]. The samples were heated for a maximum of 30 min above denaturation temperature and for 45 min below denaturation temperature.

### 2.4. Size-Exclusion Chromatography Coupled with Fluorescence Intensity Detection

The denaturation of patatin and the formation of aggregates, as well as the interaction with protease inhibitors during the heating experiments, were investigated by size exclusion chromatography (SEC). The separation was performed on an Agilent 1100 Series chromatograph (Agilent Technologies, Waldbronn, Germany) equipped with a quaternary pump. The system was controlled by Agilent ChemStation software (Rev. C.01.08). An analytical guard cartridge (GFC 4000 4 × 3.0 mm) was used. The SEC column used was a “Yarra 3 µm 7.8 × 300 mm SEC 4000” column (Phenomenex, Torrance, CA, USA). For eluting the protein from the column, a 100 mM sodium phosphate buffer decontaminated with dimethyl dicarbonate (DMDC) with a flow of 1 mL/min was used. The protein solution was mixed 1:1 with the elution buffer, filtered through a 0.45 µm syringe filter (Chromafil Xtra RC, Düren, Germany), and injected onto the column. The elution signal was determined at 214 nm with a diode array detector (DAD). To calibrate the retention times to certain molecular weights, a commercial SEC calibration standard (Phenomenex, Torrance, CA, USA) with proteins ranging from 0.214–900 kDa was used. (A sample chromatogram of the SEC standard can be found in Supplementary Materials Figure S2). Monomeric patatin has a molecular mass of around 40 kDa [7] to 43 kDa [27]. Native dimers [27] and trimers [28], with 80 and 120 kDa, are described in the literature. Therefore, the UV signal between 44 and 150 kDa was described to the patatin fraction in the protein solution. The fraction with a molecular weight below 44 kDa was ascribed to the protease inhibitor (PI) fraction. The molecular weights of these proteins are typically 25 kDa and below [25]. To investigate the intrinsic fluorescence of protease inhibitors, patatin, and the formed aggregates, a fluorescence detector (FLD) was added after the diode array detector. Samples were excited at 280 nm, and the emission spectrum in the range of 300–400 nm was recorded 8a sampel FLD chroamtogram can be found in the Supplementary Materials Figure S3. By injecting different aliquots of a solution prepared with the patatin standard described above, the area of the elution signal obtained by SEC could be attributed to a certain amount of protein. A sample chromatogram can be found in the Supplementary Materials Figure S4.

### 2.5. Determination of Free Thiol Groups in Heated PPI Solutions

The loss of free thiol groups during the heat-induced aggregation of patatin was measured through the reaction of thiol groups with the reagent 4,4′-Dithiodipyridine (DTDP). Free thiol groups crosslink in the presence of the dye and result in the conversion of DTDP into 4-thiopyridine (4-TP). 4-TP has an absorption maximum at 324 nm and can be quantified on a reversed-phase high-pressure liquid chromatography system (RP-HPLC) coupled with a DAD. The amount of 4-TP formed is directly related to the amount of free thiols in a sample. The absorption signal was calibrated through calibration with cysteine in different concentrations. A detailed description of sample preparation, evaluation, and method validation can be found elsewhere [29].

To calculate the theoretical amount of free thiols in the aggregates, Equation (1) was used;

(1) $$\frac{C_{Pat,SEC}}{M_{Pat}}=n_{Thiol,Pat}$$

with *C_Pat,SEC_* being the concentration of native sized patatin obtained by SEC in g/L, *M_Pat_* the molecular weight of Patatin (40 kDa) and *n_Thiol,Pat_* the molar concentration of free thiols in the solution in mol/L. The molar concentration is calculated under the assumption that each native sized patatin molecule only contains one free thiol group [10,11].

This thiol concentration (*n_Thiol,Pat_*) can be subtracted from the total amount of thiols determined by the RP-HPLC method (*n_total,Thiols_*) (see Equation (2)). In this way, an excess amount of thiols was obtained (*n_Thiols,excess_*)

(2) $$n_{total,Thiols\;}-n_{Thiol,Pat}=n_{Thiols,excess}=n_{Thiol,Aggregates}$$

An excess of thiols indicates that more thiols are measured in the solution than would be predicted by the amount of native sized patatin. Therefore, changes in *n_Thiols,excess_* must occur within the protease inhibitor or the aggregate fraction. As will be shown in this study, the interaction of protease inhibitors with the patatin is low. Therefore, we assume that excess thiols are mainly measured due to the fact that reactive thiols reside within the aggregate fraction (*n_Thiol,Aggregates_*).

However, the calculated amount should not be viewed as a quantitative measurement. It is rather a qualitative description of how the protein denaturation, measured by SEC, correlates with the loss of free thiol groups. If the loss of free thiols moves in tandem with the denaturation and subsequent aggregation of patatin, the value will not change much. If denaturation and aggregation are much faster than the loss of free thiols, the calculated amount of free thiols in the aggregates will increase.

### 2.6. Blocking of Protein Interactions

To investigate the contribution of different protein interactions on the aggregation behavior of PPI, protein solutions were heated in the presence of sodium dodecyl sulfate (SDS), N-Ethylmaleimide (NEM), urea, and Tween 20. Each of these are able to block certain protein interactions [17]. For this, 2.2% PPI solutions were prepared in distilled water and stirred overnight. After stirring, the solution was centrifuged at 6000× *g* for 15 min to eliminate undissolved particles. The protein concentration of the supernatant was around 2%. The 2% solution was mixed in a 1:1 ratio with the blocker substances to obtain final concentrations of 1% (*w*/*w*) SDS, 20 mmol/L NEM, 3 mol/L urea, and 0.5% (*w*/*w*) Tween 20. The solutions were heated for 30 min in a heated shaker (HLC—Haep Labor Consult, Bovenden, Germany) at 10 °C above denaturation temperature to ensure total denaturation of the proteins. The solutions were then analyzed by gel electrophoretic analysis as described below. A sample PAGE can be found in the Supplementary Materials Figure S5.

### 2.7. Gel Electrophoretic Analysis

To investigate the formation of covalently linked aggregates and the loss of patatin during heating, polyacrylamide gel electrophoresis (PAGE) was utilized. Therefore, the protein solution was diluted to 2 mg/mL protein in two types of buffers. One buffer containing 10% SDS, 0.5 M Tris-HCl, and 0.5% bromophenol blue pH 6.8 for non-reducing condition and another buffer containing 10% SDS, 0.5 M Tris-HCl, 0.5% bromophenol blue, and 15 mg/mL dithiothreitol (DTT) pH 6.8 for reducing condition. Samples were heated at 100 °C for 5 min to allow for complete interaction with SDS. For analysis, a prepacked, stain-free TGX gradient gel (4–20%) (Bio-Rad Lab., Hercules, CA, USA) was used. Each well of the gel was loaded with 10 µL of sample dissolved in buffer. A standard marker Precision Plus Protein^TM^ Standard (Bio-Rad Lab., Hercules, CA, USA) was loaded on a separate well. Protein separation was run at 300 V, 50 mA/gel, and 35 W for approximately 35 min. Protein bands were made visible at 300 nm and scanned using Molecular Imager Gel Doc^TM^ XR system (Bio-Rad Lab., Hercules, CA, USA) controlled with ImageLab (v 6.0) software. The protein bands were assigned as follows:

The band intensity around 40 kDa, was ascribed to monomeric patatin in the sample. A second band around 100 kDa could be some residual enzyme-like lipoxygenase present in PFJ [6]. The bands at 25 kDa and below were ascribed to the protease inhibitors present in the protein solution. Under non-reducing conditions, the 100 kDa band can also consist of dimeric patatin. Prolonged heating can lead to an increase in band intensity above 100 kDa, due to the formation of bigger aggregates.

### 2.8. Determination of Exposed Hydrophobicity

A 1.41 mM *N*,*N*-dimethyl-6-propionyl-2-naphthylamine (PRODAN) solution was prepared by dissolving PRODAN in pure methanol to ensure complete dissolution of the fluorescence probe. The solution was kept at −40 °C under the exclusion of light. For each measurement, small aliquots of the sample were taken from the freezer. Aluminum foil ensured the exclusion of light from the solution, and it was kept over ice for the whole time. Protein solutions were diluted to 1 mg/mL. 1 mL of the diluted solution was transferred into a deep well plate. The PRODAN solution was added in amounts of 0, 5, 10, 15, 20, 25, 30, 40, and 50 µL. Afterward, the solution was thoroughly mixed with a pipette. 100 µL of the mixed solutions were transferred to a black 96-well plate (Greiner chimney flat back 96 well), and the mixture was incubated for 30 min at room temperature in the dark. All solutions were transferred in triplicate onto the plate. After incubation, the solutions were excited at 365 nm in a Tecan Spark microplate reader (Tecan Group Ltd., Männedorf, Switzerland). The emission spectra between 400 and 650 nm were recorded. A maximum was detected at 440 nm, and the absorption value at this wavelength was plotted against the amount of PRODAN added. For most solutions, 30 µL marked the beginning of a plateau in the fluorescence intensity indicating saturation of all hydrophobic binding sites. The fluorescence intensity over the amount of PRODAN in the linear region before the plateau was fitted by linear regression, and the slope of regression was taken as a measure of the exposed surface hydrophobicity (S_0_).

### 2.9. Data Evaluation and Kinetic Data Fit

Experiments were done in duplicate from two independently prepared solutions. If not described otherwise, the depicted data points describe the average of two measurements and error bars the min/max range of the measurements. As experiments were only done in duplicate, no standard deviations could be obtained. The kinetic data of the loss of native patatin during heating was obtained from the SEC experiments described above. To derive kinetic parameters from the SEC data, the loss of native patatin at every point in time (*C_t_*) in relation to native patatin in the unheated sample (*C*_0_) was fitted by non-linear regression (see Equation (3));

(3) $$\frac{C_{t}}{C_{0}}={\left[\left(n-1\right)k_{app}*t+1\right]}^{\frac{1}{1-n}}\;\mathrm{for}\;n\;\neq \;1$$

where *n* is the reaction order and *k_app_* is the apparent rate constant. The formula was already used by other research groups [10,30] for the fitting of kinetic data.

## 3. Results and Discussion

### 3.1. Heat-Induced Denaturation and Aggregation in PPI Solutions

**Figure 1:**
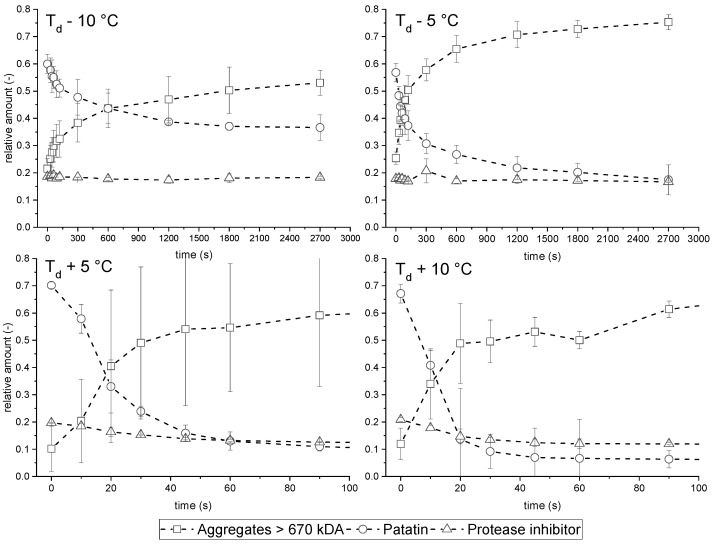
Change of absorbance area measured by SEC of the different protein fractions, exemplarily shown for a 1% PPI pH 7 solution after heating for different durations. As denaturation above T_d_ happens very fast, only the first 100 s of are depicted.

Heating a protein solution for a prolonged time results in the unfolding of the proteins and subsequent formation of higher molecular aggregates. The denaturation of a pH 7 PPI solution can be seen in Figure 1. The biggest changes occurring during heating relate to the loss of native size patatin and the increase of the aggregate fraction (>670 kDa). Smaller aggregates are also present in the protein solution. However, these readily react into bigger aggregates (>670 kDa), as was already observed by others [8].

It can be seen that the loss of native patatin goes in parallel with the increase of the large aggregate fraction. It is, therefore, reasonable to assume that the formed aggregates mainly consist of aggregated patatin. Furthermore, it can be seen that the PI fraction is unaffected by the heat treatment. Although PI was shown to unfold and form gels at temperatures around 70 °C [16], nearly no interaction between PI and patatin could be seen. Small decreases in the protease inhibitor fraction were observed for the higher temperatures investigated, which will be explained later when the intrinsic fluorescence spectra of the solution are discussed.

### 3.2. Kinetic Parameters of Patatin Denaturation and Aggregation

**Figure 2:**
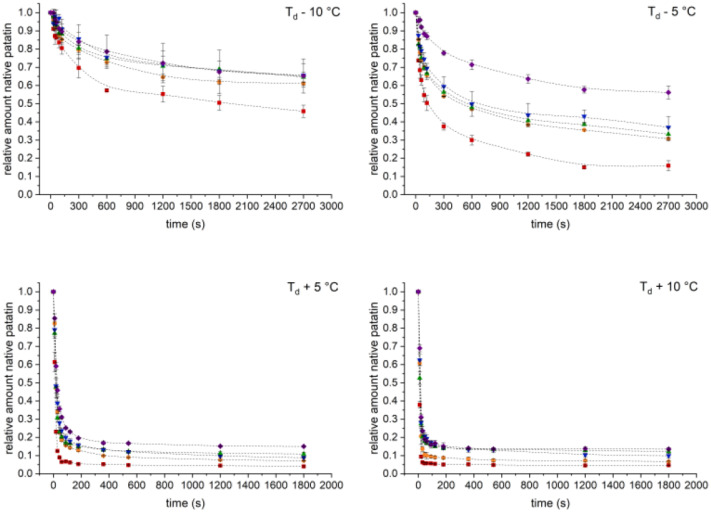
Decrease of native sized patatin in relation to the unheated sample during heating, determined by SEC.

Prolonged heating of the PPI solutions resulted in the loss of native-sized patatin due to unfolding and aggregation (Figure 2). When heating was done above T_d_, the loss of patatin was fast, and residual native-sized protein was below 20% within 200 s. When heating was done below T_d_, the loss of native patatin was much slower; however quite similar for pH 7, 8, and 9. The most pronounced differences between the denaturation curves were found at the extremes of the investigated pH range. The fastest denaturation occurred for PPI solutions at pH 6, and the slowest denaturation was observed for pH 10. This showed that electrostatic repulsion has an important influence on the aggregation behavior of patatin. The reduced electrostatic repulsion at pH 6 increases the likelihood of the unfolded protein monomers aggregating, whereas the in-creased electrostatic repulsion at pH 10 decreases aggregation. Because of this, the aggregation is slowed for alkaline conditions.

Furthermore, the loss of native protein in a solution can be used to derive reaction parameters of the underlying chemical reaction. One simple method for fitting kinetic data is through non-linear regression, demonstrated for a variety of β-lg data [30] as well as for aggregation kinetics of ovalbumin and patatin [10].

**Figure 3:**
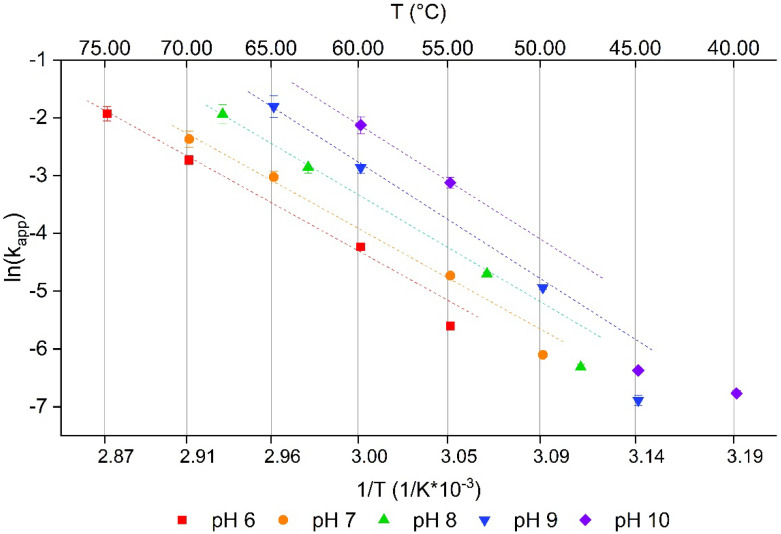
Arrhenius diagram of the PPI denaturation reaction rate. The reaction rate is obtained by fitting Equation (3) to the data presented in Figure 2. For temperatures 10 °C below the respective denaturation temperature, a deviation from the linear behavior is observed. The dashed line is meant as a guide for the eye.

From the denaturation described in Figure 2, the apparent reaction rate (k_app_) can be derived through non-linear regression. From the reaction rate, the Arrhenius diagram of the reaction can be obtained, as shown in Figure 3. The reaction orders of the PPI aggregation in dependence of the denaturation temperature and the pH are given in Table 2. For all pH values, a decline in reaction order was observed with increasing temperatures. For comparison, data on patatin denaturation at pH 7 [8] was fitted with the same non-linear regression, and a similar trend could be seen. At 50 °C, a high reaction order of 8.78 ± 2.44 was found, which decreased to 2.39 ± 0.14 at 65 °C. Another study reported a reaction order of 2.9 when fitting the average of patatin denaturation data under different heating conditions [10]. However, in this study, certain conditions clearly deviate from the averaged trend line, something which was not observed for the other proteins (β-lactoglobulin and ovalbumin) in the study. For example, denaturation at low temperature and/or low electrostatic repulsion behaved very differently from denaturation at higher temperatures and at neutral pH. Therefore, changes in the reaction order appear to be more pronounced for patatin than for β-lg.

Figure 3 shows the Arrhenius diagram of the denaturation kinetics of PPI. A change in reaction rate in dependence of the temperature of unfolding can be seen. The k_app_ of the three highest temperatures investigated for each pH value, with the exception of pH 10, are on a straight line. The lowest investigated temperature exhibited the lowest k_app_ and deviated clearly from the trend line of the other three data points. This could indicate two different ways on how the proteins unfold and aggregate.

For β-lg, a bend in the Arrhenius diagram was already observed for purified protein [31] and in different milk milieus [32]. The reaction at lower temperatures was described as rate limited by the unfolding step and for higher temperatures as rate limited by aggregation. Under unfolding limited conditions, not every protein monomer is unfolded and therefore reactive. Therefore, not each collision of two proteins results in aggregation. Furthermore, when the temperature was not too high and irreversible aggregation not likely, proteins can also refold. The fact that patatin is able to refold was already observed by others [7]. Contrary to this, the protein collisions result in high rates of irreversible aggregation in case most proteins are already unfold-ed. For β-lg, this is the case when the temperature is sufficiently high so that all proteins in a solution are unfolded. This was found to be the case around 90 °C, as analytically determined by DSC [31].

This is a clear difference from what was found in this study for patatin. The bend in the Arrhenius diagram for patatin occurs somewhere between T_d_ − 10 °C and T_d_ − 5 °C. At T_d_ − 5 °C, less than 20% of protein is unfolded according to the DSC data (data not shown). This indicates that already small amounts of unfolded or partially unfolded protein are enough to change the aggregation kinetics towards diffusion rate-limited kinetic behavior. This might also explain why aggregation of patatin is much faster than β-lg [10]. Due to the lack of internal disulfide bonds, patatin’s tertiary structure is less stabilized than β-lg. Therefore, unfolding in patatin should happen at a higher degree and the exposure of hydrophobic groups is more pronounced. The fact that native patatin already develops considerable interactions with hydrophobic interaction columns [11] gives an indication that this protein, even in its native state, might be more readily prone to aggregation driven by hydrophobic interactions. As the aggregation should be mainly initiated through exposed hydrophobic groups, these groups might initiate aggregation even with native proteins, allowing for diffusion-limited aggregation despite not all proteins being unfolded. The reason why pH 10 deviates from this trend is probably due to the low temperatures used for denaturation. Furthermore, the high negative charge at such elevated pH levels might lead to the unfolding of proteins independent of heat application, influencing the overall aggregation mechanism. In addition, aggregates could dissolve and form denatured monomeric proteins in extreme alkaline conditions. For example, whey protein aggregates at pH levels of 11 and above were shown to dissolve [33].

At a given temperature, decreasing the pH resulted in decreases in the reaction rate, and increases in pH resulted in increases in the reaction rate [10]. However, it is known that lowering the pH towards the IEP of a protein results in an increase in heat-resistance of the protein, seen in in-creased T_d_. The reason for this are stronger intramolecular interactions between the amino-acid residues due to a reduced electrostatic repulsion between the chains [34]. Therefore, when heating temperatures are set in relation to the T_d_, the lower electrostatic repulsion between the proteins facilitates protein aggregation.

### 3.3. Protein–Protein Interactions within PPI Aggregates Measured by SDS-PAGE

**Figure 4:**
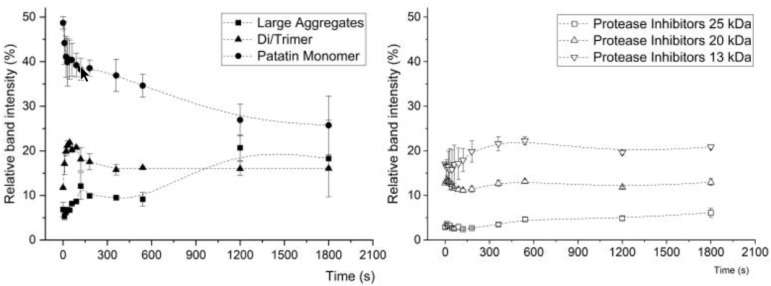
Change in protein amount of the patatin and aggregate fractions of a 1% PPI solution at pH 7 in dependence of the time heated at 70 °C. The band intensity was averaged from two SDS-PAGE gels under non-reducing conditions, and the range is indicated by the error bars.

By applying reducing and non-reducing SDS-PAGE, the formation of covalently linked protein aggregates can be investigated. To determine how the formation of covalent bonds changes during thermal aggregation, a PPI solution with pH 7 was heated at 70 °C, and samples were taken at different time steps, see Figure 4. Under non-reducing conditions, a decrease in monomeric patatin and an increase in di- and trimers and larger aggregates were detected. This showed that prolonged heating of patatin results in more disulfide bond formation between the monomers. When the heated solutions under reducing conditions were investigated, the native-sized patatin fraction had a similar band intensity as in the unheated sample.

Furthermore, the protease inhibitor fraction showed no pronounced changes in band intensity over the heating time. This led to the conclusion that the protease inhibitor fraction does not participate in aggregate formation via covalent bonds.

**Figure 5:**
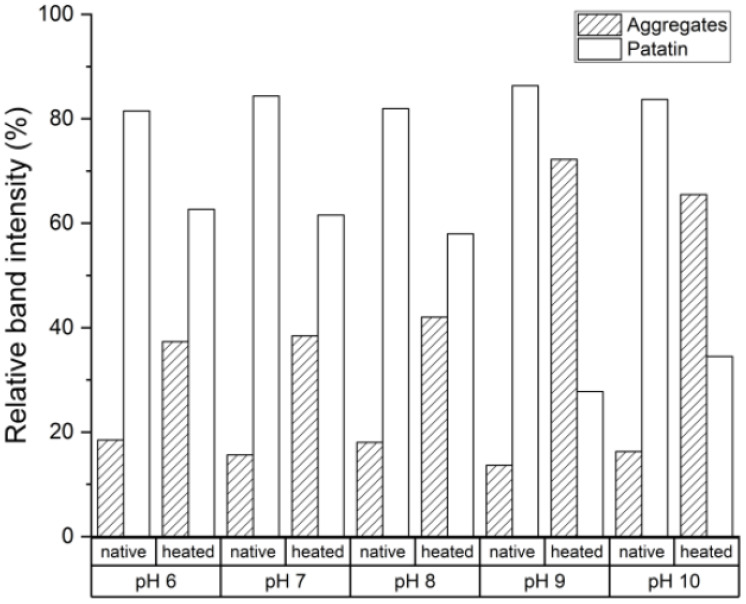
Influence of pH on the amount of native sized and aggregated patatin in dependence of the pH in a non-reducing SDS-PAGE. The heating was done 10 °C above T_d_ for 30 min. The band intensity was taken from one SDS-PAGE gel under non-reducing conditions.

For other protein systems, like whey and egg proteins, alkaline pH levels led to increased reactivity of thiol groups and subsequently to more disulfide bond formation [2,35,36]. The main difference between these protein systems and the patatin system is that patatin lacks internal disulfide bonds. Therefore, a thiol-disulfide exchange reaction is not likely to occur. Nevertheless, disulfide-linked trimeric structures were described for very diluted PPI solutions heated at neutral pH [9]. The aggregation mechanism behind these trimeric structures remained unclear. In Figure 5, it can be seen that the amount of monomeric patatin decreased for all pH values upon heating. Furthermore, increasing the pH value to alkaline levels resulted in patatin forming more covalently linked aggregates. This is a clear indication that patatin is able to form disulfide-linked aggregates and that the reactivity of the thiol group is increased with alkaline pH values. At first, the increased aggregate formation at alkaline pH levels seems like a contradiction to what was described for the SEC results where lower amounts of aggregates were found at alkaline levels. However, one has to consider that the SEC measures the protein solution in an aqueous phosphate buffer. Therefore aggregates linked through non-covalent bounds are intact. In the SDS buffer, only covalent linked aggregates are shown. It can be concluded, that at alkaline pH values aggregation in PPI is slowed down compared to neutral or slightly acidic pH. However, the aggregates that do form are rather formed through disulfide bonds than non-covalent bonds. The reactivity of the free thiol groups will be described in detail further below.

**Figure 6:**
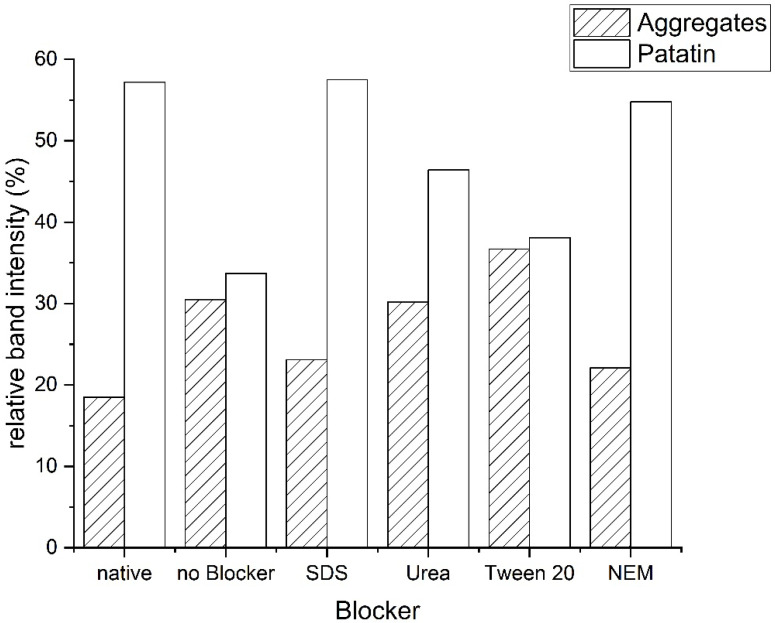
Relative amount of protein in the aggregate and patatin fraction under non-reducing conditions in the presence of different blocking substances. The heating was done 10 °C above T_d_ for 30 min. The band intensity was taken from one SDS-PAGE gel under non-reducing conditions. The solution heated in the presence of NEM formed a weak gel. This indicated a strong influence of the thiol groups on the aggregation behavior of PPI.

For a better understanding of the protein interactions forming the aggregates, PPI solutions were mixed with the blocking substances sodium dodecyl sulfate (SDS), N-Ethylmaleimide (NEM), urea, and Tween 20. Upon heating, the relative intensity of the patatin and the aggregate fraction under non-reducing conditions changed (see Figure 6). The reduction in patatin indicated the formation of covalently linked aggregates from this protein. The formation of covalently linked aggregates can also be seen when the aggregate fraction is increased upon heating. Non-covalently linked proteins are dissociated by the SDS-PAGE buffer and can, therefore, not be detected.

Tween, with its large non-polar structure, is expected to disrupt hydrophobic protein interactions [37]. Urea is described as a blocker of hydrogen bonds [38] as well as a blocker of hydrophobic interactions [39,40,41]. For both systems, the band intensity of the aggregate peak was reduced, and patatin was increased compared to the heated sample without added blocker. This indicates that if the non-covalent interactions are inhibited to a certain degree, the formation of covalent disulfide bonds is reduced as well.

That initial interactions through hydrophobic interactions are necessary for the formation of disulfide bonds is even more apparent when SDS is used as a blocker. SDS binding to proteins correlates very well with the hydrophobicity of the protein [42,43,44]. Therefore it is used to investigate the contribution of hydrophobic interactions in the aggregation and gelation of protein systems [17,38,45,46]. SDS prevented the formation of disulfide-linked aggregates, as indicated by a similar band intensity of the heated patatin band as in the unheated sample. This indicates that at first, a hydrophobic interaction between protein monomers has to occur before disulfide links can be formed. This was also proposed by other researchers investigating the aggregation of β-lg [47,48].

The fact that disulfide bridges were indeed involved in the formation of aggregates was additionally shown through the addition of NEM. NEM binds to the free thiol groups available in the protein solution and should therefore inhibit any disulfide formation. This led to the smallest measured aggregate fraction and the same amount of native-sized patatin measured as in the unheated reference.

It can be concluded that patatin rich potato protein isolate can form aggregates that are intermolecularly linked by disulfide bonds. However, these disulfide-linked aggregates are around 250 kDa and smaller, in the presence of SDS; this is considerably smaller than the size of aggregates measured by SEC. The aggregates in the SEC had a size of over 1.500 kDa, which is the size exclusion limit of the used SEC column. Therefore it can be concluded that smaller disulfide-linked aggregates (around 250 kDa) interact through non-covalent interactions to form bigger aggregates of over 1.500 kDa.

Based on these results, the importance of disulfide links within the aggregates is apparent when taking a closer look at the aggregation in the presence of NEM. The 1% PPI solution in 20 mM NEM gelled upon heating. This behavior was not observed for any of the other solutions in the presence or absence of blocking substances. Gelation at 1% protein concentration is considerably lower than the 6% minimum gelling concentration reported before for patatin [11]. The blocking of the thiol group might lead to more exposure of hydrophobic groups that induce the gelation, something which was observed for β-lg and soybean protein heated in the presence of NEM [49,50,51].

To sum up, disulfide links were not shown to induce polymerization in patatin, the same way as reported for β-lg. Nevertheless, blocking of thiols seriously altered the techno-functional properties of the aggregates. Therefore, the reaction of thiols during heat-induced aggregation of PPI will be investigated in more detail in the following.

### 3.4. Reduction of Free Thiol Groups in PPI Solutions and Formation of Disufide Bonds during Heat-Induced Aggregation

**Figure 7:**
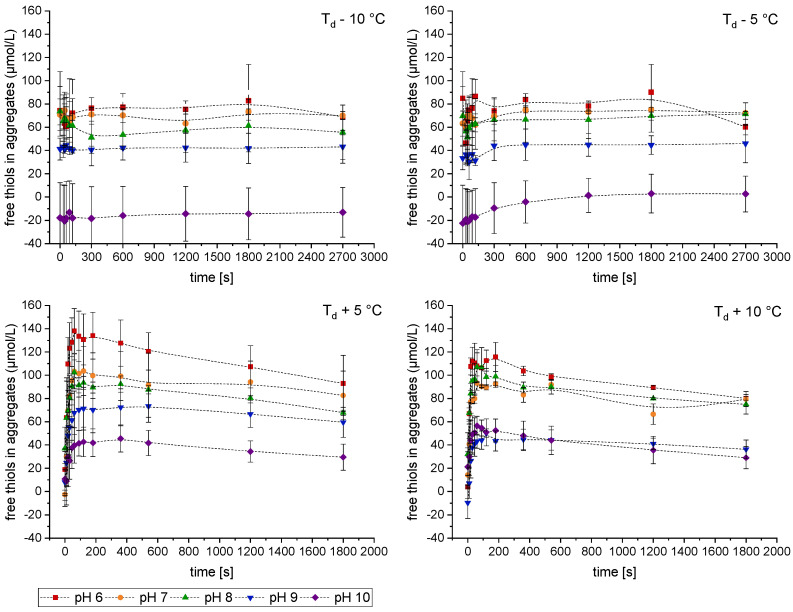
Amount of free thiol groups in the aggregate fraction during heating of a PPI solution in dependence of temperature and pH. Error bars represent the range of two independent heating trials.

The loss of free thiols during heating was correlated with the denaturation of the native-sized patatin fraction. The data was processed according to Equations (1) and (2) to obtain the theoretical amount of thiols in the aggregate fraction. The results of this comparison are shown in Figure 7. Higher pH values resulted in more disulfide formation, thus lowering the amount of free thiols within the aggregates. Negative values indicate that less thiol groups are measured than could be expected from the amount of native sized patatin. As SEC measures also dimers as native sized patatin disulfide bonds that links dimer without being incorporated in bigger aggregates will lead to a negative value.

For all investigated temperatures, the solutions at pH 6 exhibited the highest amount of free SH groups within the aggregates. This can be explained by the electrostatic interactions influenced by the pH level. At reduced electrostatic repulsion, protein molecules can interact with each other more readily. Furthermore, the formation of short-ranged non-covalent bonds like hydrogen bonds is increased. In a study on PPI gels, it was shown that closer to the IEP, the relative contribution of electrostatic and hydrogen bonds to the gel network was higher than for neutral or alkaline pH [26]. When patatin aggregates at low electrostatic repulsion, the aggregation is fast. Furthermore, aggregates grow in size, turning the solution turbid. The proteins interact mainly through non-covalent bonds, and there is a low amount of disulfide formation.

The temperature also has a strong influence on the formation of disulfide bonds. Below T_d_, the aggregation was slow and limited by the unfolding of the proteins. At the lowest investigated temperatures (T_d_ − 10 °C), patatin denaturation and loss of free thiols moved in tandem. Under these conditions, each patatin molecule that unfolds and aggregates forms a disulfide bridge. Therefore, no excess free thiols in the aggregates were measured, and the calculated amount of free thiols in the aggregate fraction remained constant. With temperatures above T_d_, the aggregation process changes. Above T_d_, most monomers are unfolded and can readily interact through exposed hydrophobic groups. The aggregation is fast and only limited by the diffusion of the proteins towards each other. For patatin heated above T_d_, a high amount of free thiols within the aggregates was detected. Therefore, an increase in reaction rate favors the formation of non-covalent bonds over disulfide bonds. However, with prolonged heating at higher temperatures, the amount of free thiols decreases, indicating that after initial aggregation through non-covalent bonds, disulfide bridges are formed. This two-step process, which involves initial aggregation via non-covalent bonds, followed by stabilization through internal disulfide bonds, was also proposed for ovalbumin [52]. Ovalbumin was shown to have similar reaction rates as patatin [10], which might be explained by a similar reaction mechanism. Besides the formation of disulfide bonds, the accessibility of hydrophobic amino acids also plays an important part in the aggregation of patatin. Therefore, the hydrophobic character of PPI during heat treatment shall be accessed in the following.

### 3.5. Change in the Hydrophobic Character of PPI Aggregates during Heat-Induced Aggregation

**Figure 8:**
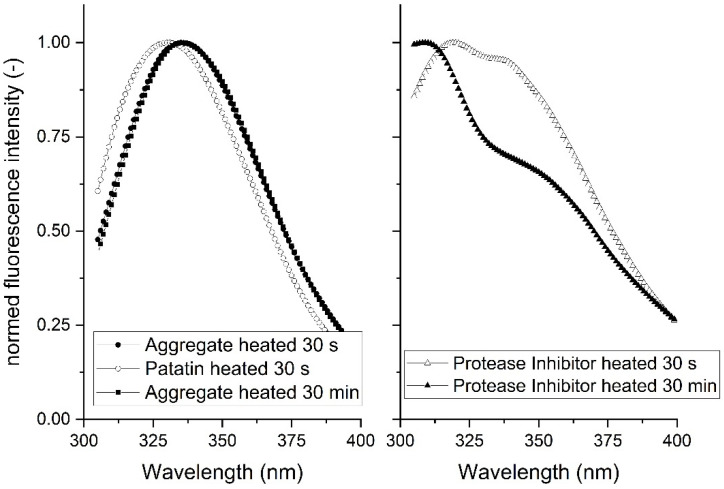
Fluorescence spectra of the patatin, aggregate (>670 kDa), and protease inhibitor fraction, determined by coupled SEC and FLD. Spectra were normed on the maximum detected emission value. Excitation of the sample was done at 280 nm.

The SEC coupled with FLD was used to investigate the local environment of aromatic amino acid groups in the different protein fractions. In Figure 8, the emission spectra of the patatin and the >670 kDa aggregate fraction are displayed. The native patatin fraction exhibited an emission maximum at around 331 nm, whereas for the aggregate fraction, a maximum was found at 341 nm. At 280 nm, both aromatic residues, tryptophan (trp), and tyrosine (tyr) can be exited. After falling back to the unexcited state, photons of a higher wavelength are emitted. The measured emission spectrum, especially the maximum, is dependent on the local environment of the excited aromatic amino acid. When the proteins are in their native state, the aromatic amino acids reside within the protein core, and the environment is mostly apolar. Upon unfolding, the amino acids are exposed to the polar water environment resulting in a shift of the emission spectrum towards higher wavelengths. This behavior has been reported for β-lg [53] and patatin [7]. The investigation of thermally induced aggregation of patatin showed a substantial refolding of the tryptophan residues when the hot solution was cooled down to ambient temperature. However, a partial shift suggested that the local environment did not fully fold back into the native state or that not all proteins did refold. Unheated patatin, as well as heated patatin samples, exhibited no difference in the fluorescence spectra (data not shown). Therefore, it can be assumed that the native–sized patatin in heated samples was either not unfolded or was refolded upon cooling. A difference between the aggregates heated for 30 s or 30 min could not be observed, Therefore, increased unfolding after longer heating times could not be detected.

From SEC and SDS-PAGE results, it could be shown that the protease inhibitor fraction does not decrease considerably for the investigated temperature and pH conditions. However, a small decrease could be observed for samples heated at high temperatures, indicating the aggregation of certain protease inhibitors. The protease inhibitor, which is the least affected by the heat treatment, had a retention time of around 11.7 min, and the fluorescence spectrum is given in Figure 8. The described protease inhibitor shows a clear fluorescence maximum around 310 nm. It is reasonable to assume that this protease inhibitor is the potato cysteine protease inhibitor (PCPI). PCPI is the second most abundant protease inhibitor and possesses trp and tyr fluorescence with a peak maximum around 310 nm [15]. With a determined unfolding peak temperature of 67 °C, PCPI should be more heat resistant than patatin, which would explain why PI aggregation plays a minor role in the investigated PPI system.

**Figure 9:**
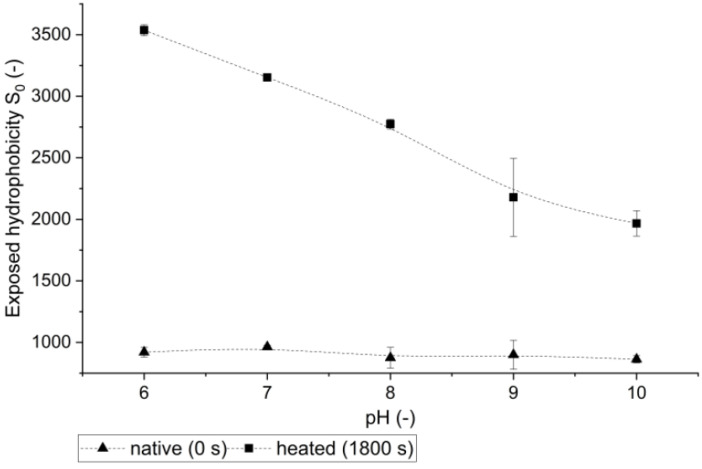
Exposed hydrophobicity of a 1% PPI solution native (unheated) and heated for 1800 s above T_d_, in dependence of the pH.

Although the unfolding of patatin aggregates was shown by the intrinsic fluorescence tests, no changes between the pH levels could be observed by this method. To investigate the influence of pH on the extent of unfolding and accessibility of hydrophobic groups, a fluorescence test with the uncharged PRODAN dye was conducted. PRODAN binds to hydrophobic amino acid residues and forms a fluorescent complex. Contrary to other fluorescence dyes, PRODAN contains no charge- Therefore, interactions between the dye and the protein should be only hydrophobic in nature. The dye was already used to measure exposed hydrophobicity in succinylated patatin samples [54]. Independent of the pH value, all unheated protein solutions exhibited a similar exposed hydrophobicity (S_0_) (see Figure 9). Heat treatment changed the affinity of the protein solutions towards the fluorescence probe PRODAN. The higher the pH value of a solution, the lower S_0_ of the solution. This indicates that hydrophobic groups are less accessible at higher pH values. A similar dependence of PRODAN-S_0_ on the pH value was recently described for BSA [55]. At high pH values, alkaline side chains are deprotonated, which will lead to the formation of hydrophobic interactions of these side chains [56]. These newly formed hydrophobic regions can be stabilized via disulfide bonds, similar to what was shown for WPI gels [23]. At pH 6, patatin monomers exhibit a lower overall charge as this pH value is closer to the protein’s IEP. However, the charge is more evenly distributed across the protein surface, allowing for more electrostatic interactions between positively and negatively charged amino acid residues [57]. Therefore, pH values closer to the IEP allow for more non-hydrophobic protein interactions. This leads to more of the hydrophobic groups exposed into the solution and increased accessibility for the fluorescence probe.

## 4. Conclusions

The influence of protein interactions on the aggregation mechanism of patatin rich potato protein isolate (PPI) has been evaluated for different heating conditions. It could be shown that disulfide-linked aggregates can be formed when initial aggregation through non-covalent bonds is possible. At low pH, aggregates grew in size, had a high amount of exposed hydrophobic and increased the formation of disulfide bonds. Therefore, disulfide bond formation groups, and low stabilization through disulfide bonds. Increasing the pH reduced the exposure of hydrophobic residues plays an important role in stabilizing hydrophobic patches within the patatin aggregates.

This knowledge can be used to alter techno-functional properties of PPI aggregates through different heating conditions and pH milieus. For example, aggregates with a high exposed hydrophobicity will be beneficial for the stabilization of air/water [18] and oil/water interfaces [19]. However, for the application as an oleogelator, more hydrophilic particles are preferred [20]. Furthermore, the aggregates can be considered building blocks of hydrogels. The different kinetics and protein interactions that happen in PPI in dependence of the pH explain why the microstructure of PPI gels differ considerably in dependence of the pH [26]. Unordered, particulate gels are formed under conditions where the aggregation is fast and a high amount of exposed hydrophobicity is present. Under conditions of high disulfide bond formation and low exposed hydrophobicity, the resulting gels are finely stranded.

## Figures and Tables

**Table 1:** Values of the peak maximum of denaturation peaks obtained through mDSC experiments. PPI samples were heated with 2 °C/min.

pH Value	6	7	8	9	10
peak maximum (T_d_)	64.5 ± 0.2	60.8 ± 0.4	57.7 ± 0.6	54.8 ± 0.1	51.5 ± 0.6

The values are given as the average of two independent runs, and ± indicates the range of this double measurement. pH values from 6–9 are taken from somewhere else [26].

**Table 2:** Reaction order of the denaturation of a PPI solution in dependence of temperature and pH.

	pH 6	pH 7	pH 8	pH 9	pH 10
T_d_ − 10 °C	6.09 ± 0.30	8.33 ± 0.39	9.57 ± 0.58	7.75 ± 0.98	8.27 ± 0.57
T_d_ − 5 °C	3.45 ± 0.10	4.96 ± 0.11	5.37 ± 0.13	5.55 ± 0.16	6.49 ± 0.28
T_d_ + 5 °C	1.10 ± 0.24	1.89 ± 0.32	2.19 ± 0.30	2.42 ± 0.31	2.32 ± 0.31
T_d_ + 10 °C	1.48 ± 0.27	1.69 ± 0.36	2.29 ± 0.40	3.02 ± 0.46	2.60 ± 0.37

The temperature is given in relation to the determined denaturation temperature T_d_. The standard error obtained from fitting Equation (3) to the data shown in Figure 2.

## Supplementary Materials

The following are available online at https://www.mdpi.com/article/10.3390/foods10040796/s1, Figure S1: Chromatograms demonstrating the shift of size fractions in SEC analysis upon heating of PPI solutions. Figure S2: Chromatogram of the SEC standard. Figure S3: 3D-FLID Chromatogram a heated PPI sample. Figure S4: SDS-PAGE of a PPI solution heated for different time intervals. Figure S5: Heat flow recorded during a mDSC measurement of a PPI sample at pH 7. Table S1: Time intervals at which heating was stopped for further analysis.
