# Supplementary material for: Influence of pH, Temperature and Protease Inhibitors on Kinetics and Mechanism of Thermally Induced Aggregation of Potato Proteins
Source: Foods. 2021 Apr 8;10(4):796. doi: 10.3390/foods10040796 (PMC8068184; doi:10.3390/foods10040796)
Supplement: Supplementary file 1 [file foods-10-00796-s001.pdf]

Supplementary Info

Table S1. Time intervals at which heating was stopped for further analysis.

Heating time for temperatures below T_d (s)	Heating time for temperatures above T_d (s)
0	0
30	10
45	20
60	30
90	45
120	60
300	90
600	120
1200	180
1800	360
2700	540
	1200
	1800

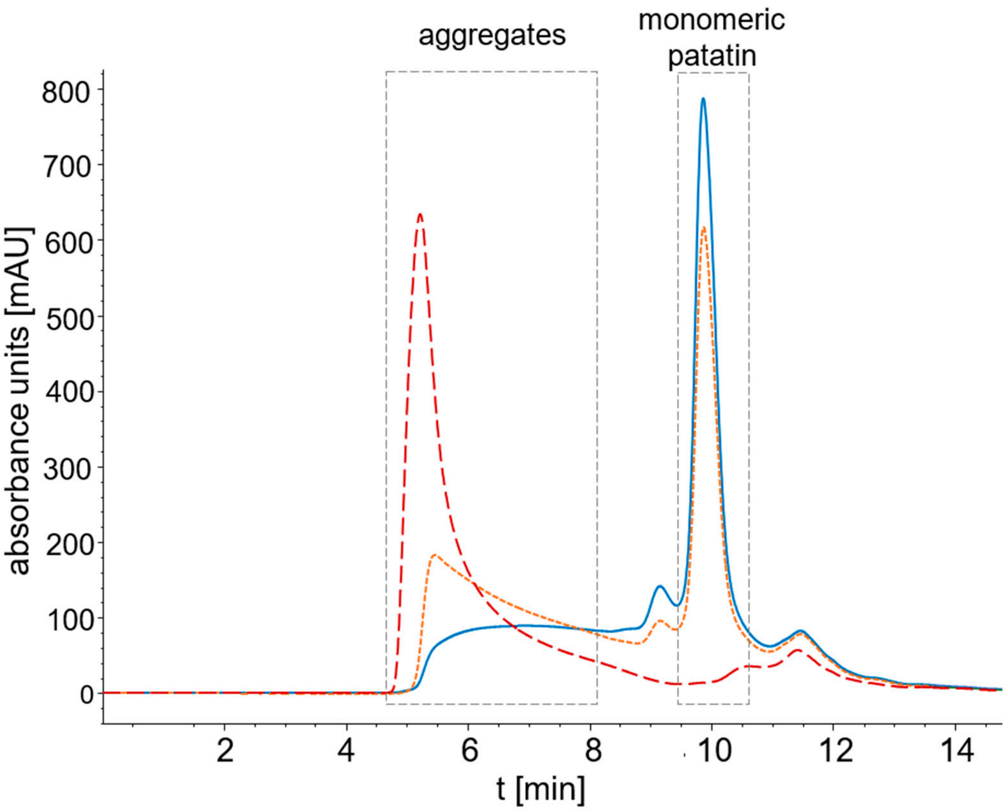

Figure 1. Chromatograms demonstrating the shift of size fractions in SEC analysis upon heating of PPI solutions. Exemplary data shown is for 1 % PPI solutions adjusted to pH 7 and heated at 65 °C (Td + 5 °C). Chromatograms shown are for PPI unheated (blue, solid), heated for 10 s (orange, narrow dashes) and heated for 30 min (red, wide dashes). Absorbance was measured at 214 nm.

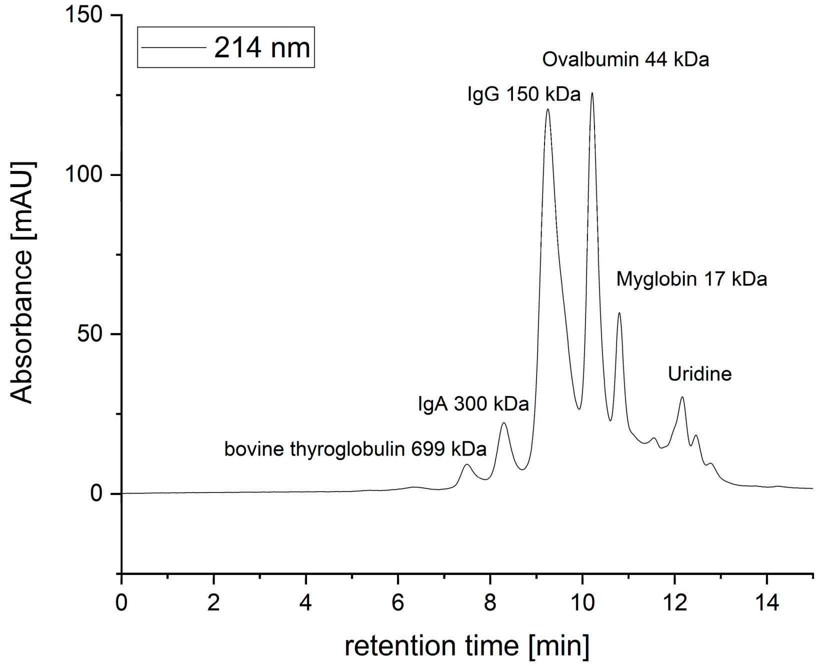

Figure 2. Chromatogram of the SEC standard. The commercial standard was applied on every test day to check the performance of the column. Absorbance was measured at 214 nm.

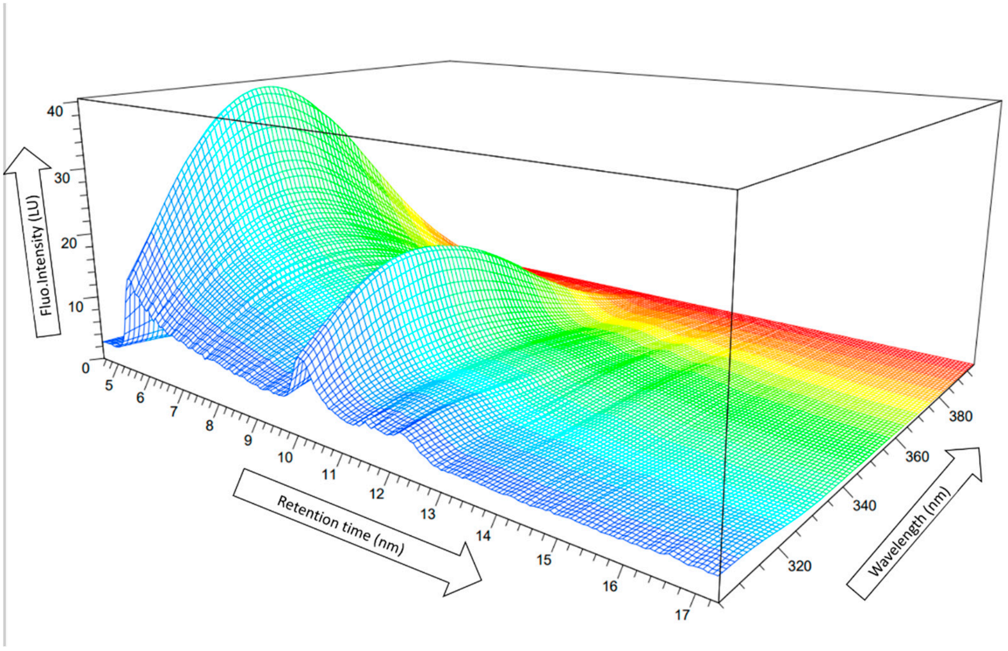

Figure 3. 3D-FLID Chromatogram a heated PPI sample. For every peak determined through the retention time the whole fluorescence spectra was recorded.

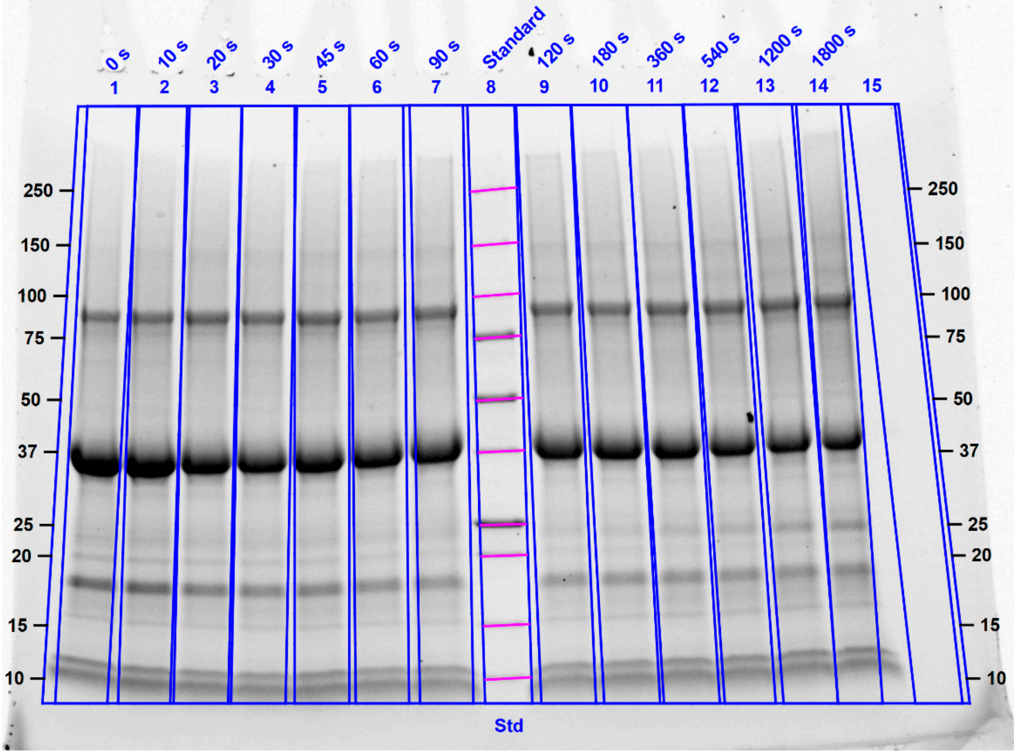

Figure 4. SDS-PAGE of a PPI solution heated for different time intervals. Protein bands at 13, 20 and 25 kDa are protease inhibitors, 40 kDa is patatin, the band around 100 kDa is containing dimers, trimers as well as a non-patatin 100 kDa fraction, 150 kDa and above is considered higher molecular aggregates. The time intervals are given on top.

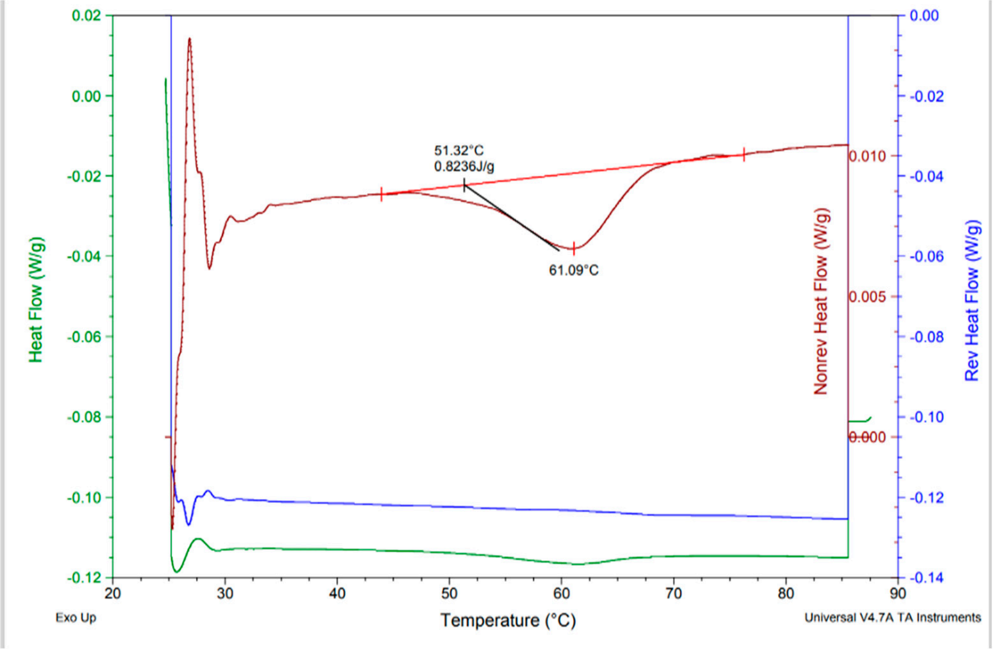

Figure 5. Heat flow recorded during a mDSC measurement of a PPI sample at pH 7. The heating ramp was set to 2°C/min. peak temperature was obtained by analyzing the non-reversed heatflow.
